# Current insight into the regulation of PD-L1 in cancer

**DOI:** 10.1186/s40164-022-00297-8

**Published:** 2022-07-30

**Authors:** Zhuandi Liu, Xibao Yu, Ling Xu, Yangqiu Li, Chengwu Zeng

**Affiliations:** 1 The First Affiliated Hospital, Institute of Hematology, School of Medicine, Jinan University, No.601, West Huangpu Avenue, Guangzhou, 510632 Guangzhou China; 2 Key Laboratory for Regenerative Medicine of Ministry of Education, Jinan University, 510632 Guangdong, China

**Keywords:** PD-L1, Cancer, Transcriptional regulation, Epigenetic regulation, Post-translational modification, Cancer immunotherapy

## Abstract

The molecular mechanisms underlying cancer immune escape are a core topic in cancer immunology research. Cancer cells can escape T cell-mediated cellular cytotoxicity by exploiting the inhibitory programmed cell-death protein 1 (PD-1)/programmed cell death ligand 1 (PD-L1, CD274) immune checkpoint. Studying the PD-L1 regulatory pattern of tumor cells will help elucidate the molecular mechanisms of tumor immune evasion and improve cancer treatment. Recent studies have found that tumor cells regulate PD-L1 at the transcriptional, post-transcriptional, and post-translational levels and influence the anti-tumor immune response by regulating PD-L1. In this review, we focus on the regulation of PD-L1 in cancer cells and summarize the underlying mechanisms.

## Introduction

The occurrence of tumors results from gene deletion, mutation, or abnormal expression in the presence of genetic and environmental factors, which eventually lead to abnormal cell proliferation. Tumor cells can be found, recognized, and eliminated by the immune system; however, the interaction between tumor cells and immunological cells is regulated by immune activating and inhibitory molecules [1]. Tumor cells inhibit the immune response by upregulating immunosuppressive chemicals and downregulating immune-activating molecules, allowing them to avoid detection and flourish. Immune checkpoints, which consist of various receptors and their respective ligands, are essential for the initiation and termination of an effective immune response [2, 3]. Immune checkpoints (ICs), such as programmed cell death protein 1 (PD-1)/programmed cell death ligand 1 (PD-L1, CD274), lymphocyte activating 3 (LAG3), cytotoxic T-lymphocyte associated protein 4 (CTLA4), hepatitis A virus cellular receptor 2 (HAVCR2, TIM3), and T cell immunoreceptor with Ig and ITIM domains (TIGIT), serve a vital role in tumor immunoevasion [4–6]. For example, high expression of myeloid leukemia cell ICs in patients with acute myeloid leukemia is associated with poor prognosis [7]. Blocking the suppressive immune checkpoint proteins with specific antibodies restores the immune system’s ability to distinguish and eliminate cancer cells and achieves favorable tumor immunotherapy results. Ipilimumab, an FDA-approved ICI, which is a monoclonal antibody directed against CTLA4, was used to treat melanoma, and since then, new drugs that regulate immune checkpoint proteins have emerged. PD-1/PD-L1 immunotherapy is promising and has recently become a hot study area in tumor immunotherapy [8]. However, the mechanisms underlying immune checkpoint blockade therapy resistance and the regulation of PD-L1 are not entirely understood. To develop a more effective and durable immune checkpoint blockade therapeutic strategy, it is necessary to understand the multiple functions and intricate regulatory mechanisms of PD-L1 in cancer. In this context, we review present knowledge about the regulation of PD-L1 in terms of transcriptional, protein, and epigenetic aspects. Furthermore, we discuss the efficient investigation of the PD-L1 regulatory mechanism.

## The expression and biological function of PD-L1

PD-L1, a PD-1 ligand, is a cell surface glycoprotein that belongs to the B7 co-stimulatory molecule family. Non-hematopoietic cells such as vascular endothelial cells and hepatocytes, in addition to hematopoietic cells, including T cells, B cells, and macrophages, constitutively express PD-L1 [9, 10]. PD-L1 upregulation occurs across a broad spectrum of cell types as a result of stimulation by pro-inflammatory cytokines and other factors, and PD-L1 is highly expressed in many cancer cells such as lung, ovarian, colon, and melanoma [11, 12].

**Fig. 1:**
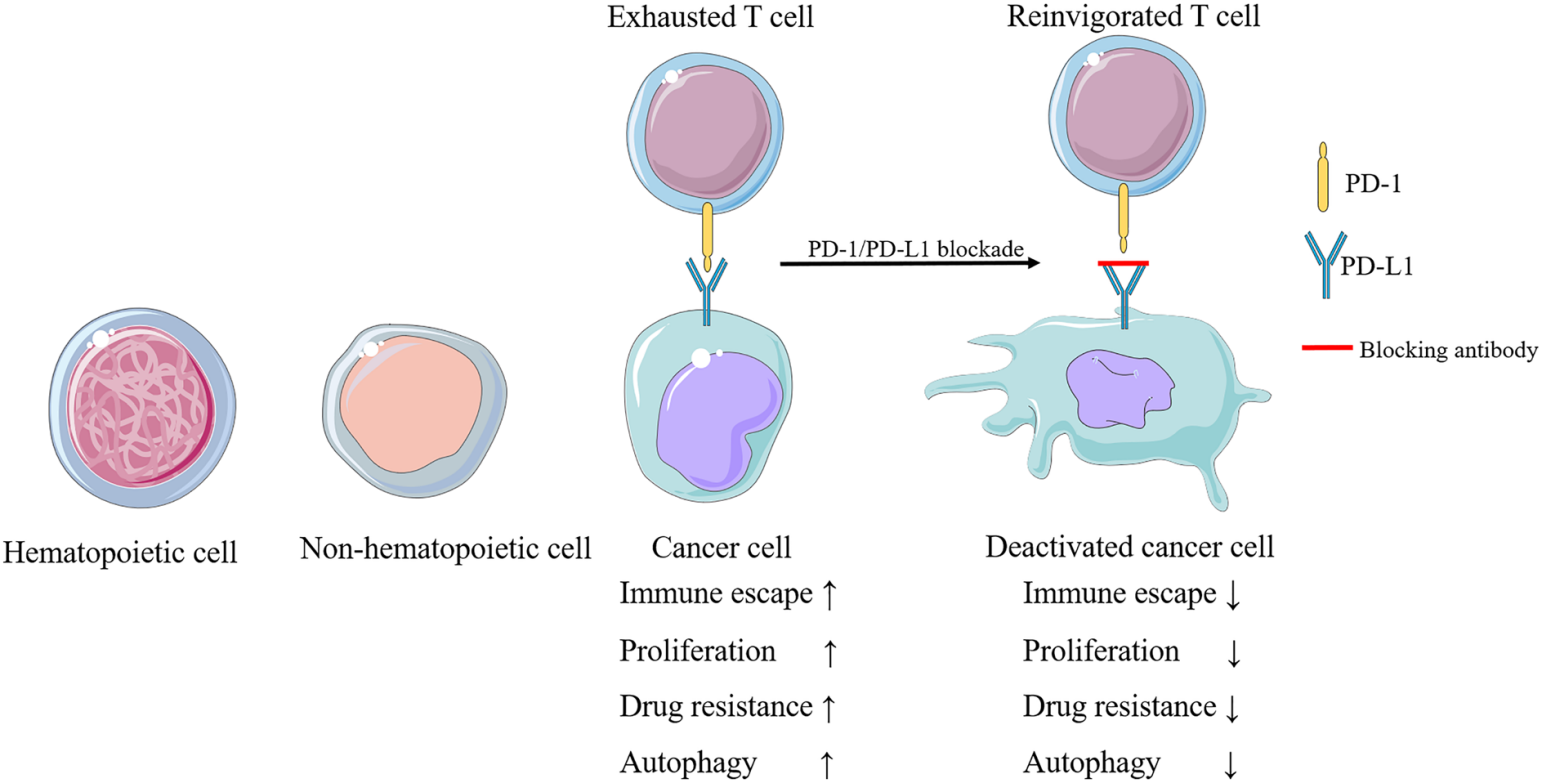
The expression and biological function of PD-L1. PD-L1 is expressed in hematopoietic cells, including T cells, B cells, DCs, macrophages, mast cells, and many non-hematopoietic cell types. PD-1 binds to PD-L1 to induce cancer cell immune escape, proliferation, drug resistance, and autophagy, and PD-1/PD-L1 blockade can inhibit these functions

Tumor cells possess abundant PD-L1, which binds to the PD-1 receptor on the surface of tumor-infiltrating lymphocytes (TILs), subsequently sending immunosuppressive signals to TILs and preventing antigen-specific CD8 + T lymphocytes from eliminating tumor cells [13–16]. Moreover, PD-L1 enables the promotion of tumor therapy through non-T cell-mediated immunity. For instance, avelumab (MSB0010718C) is a monoclonal antibody that targets PD-L1 and exhibits antibody-dependent cell-mediated cytotoxicity (ADCC), thereby significantly enhancing the killing effects of high-affinity natural killer cells in head and neck squamous cell carcinoma (HNSCC) [17, 18]. Additionally, PD-L1 is capable of promoting cancer cell proliferation. Nuclear PD-L1, coupled with transcription factor Sp1, activates the MERTK signaling pathway by regulating Gas6 mRNA synthesis and promoting Gas6 secretion, which promotes cell proliferation in non-small-cell lung cancer (NSCLC) [19]. Patients with high PD-L1 expression are more likely to have vascular invasion and tumor recurrence than those with low PD-L1 expression in rectal cancer [20]. In addition to playing a significant role in immunotherapy, PD-L1 contributes to the development of tumor drug resistance. Gemcitabine and oxaliplatin are the main chemotherapeutic agents for gallbladder cancer; nevertheless, placenta-specific protein 8 (PLAC8) can lead to the development of chemotherapy resistance through upregulation of PD-L1 expression [21]. Likewise, nuclear factor E2-related factor 2 (NRF2) promotes PD-L1 expression by inhibiting miR-1 expression, ultimately increasing the resistance of hepatocellular carcinoma (HCC) cells to sorafenib [22]. Studies have shown that radiation therapy induces systemic antitumor responses and enhances the sensitivity of refractory tumors to immunotherapy; therefore, stereotactic body radiation therapy in combination with PD-1/PD-L1 blockade may improve drug resistance in patients with advanced NSCLC [23]. The above studies suggest that PD-L1 suppresses T cell function and natural killer cells to play a role in tumor immune escape, but also promotes tumor development and tumor drug resistance (Fig. 1).

## Transcriptional regulation of PD-L1

**Fig. 2:**
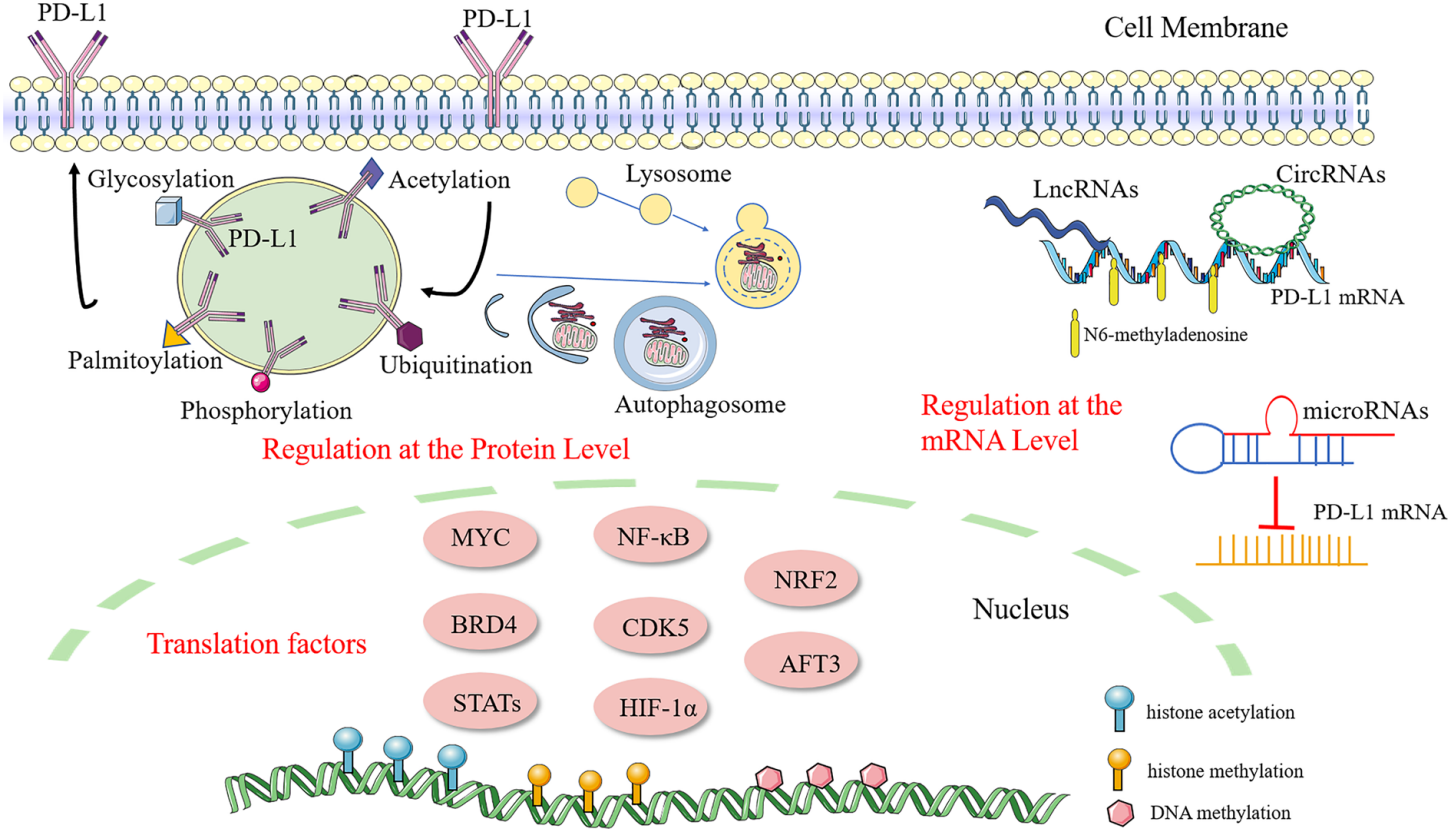
Overview of the regulatory mechanisms involved in PD-L1 expression. By attaching to the PD-L1 promoter, numerous transcription factors contribute to the increase of PD-L1 expression. N6-methyladenosine increases PD-L1 expression while DNA methylation, histone modification, and autophagy suppress it. MicroRNAs, including miR-138, miR-138-5p, miR-152, and others shown in Table 1, suppress PD-L1 by directly binding to the 3’UTR of PD-L1 mRNA. LncRNAs and circRNAs are also relevant to PD-L1 expression and tumor immune escape. PD-L1 is upregulated by glycosylation and palmitoylation, which stabilize PD-L1 protein, while ubiquitination, phosphorylation, and acetylation exert the opposite effect

Transcription factors are proteins that bind to DNA-regulatory sequences to modulate the rate of gene transcription. PD-L1 expression is regulated by a wide range of transcription factors, containing MYC, bromodomain containing 4 (BRD4), STAT family members, nuclear factor kappa-B (NF-κB), cyclin-dependent kinase 5 (CDK5), hypoxia-inducible factor 1 subunit alpha (HIF-1α), NRF2, and cyclic AMP-dependent transcription factor 3 (AFT3) [24]. Here, we summarize the critical transcription factors involved in PD-L1 expression (Fig. 2).

## MYC

MYC is a nuclear phosphoprotein involved in proliferation, apoptosis, and differentiation [25]. In a Tet-off transgenic mouse model of MYC-induced T cell acute lymphoblastic leukemia (T-ALL), PD-L1 and MYC expression were significantly correlated. Coupled with the promoter of PD-L1, MYC can directly regulate the transcription. Both inactivation and knockdown of MYC can reduce PD-L1 expression [26]. Moreover, overexpression of bridging integrator-1 (BIN1) in NSCLC could reverse PD-L1-mediated immune escape by inhibiting the expression of MYC [27]. Moreover, cell cycle protein-dependent kinase 7 (CDK7) inhibitor THZ1 could downregulate PD-L1 expression by inhibiting MYC activation, and when combined with the PD-L1 inhibitor Atezolizumab improves the outcome of NSCLC [28].

## BRD4

BRD4, a member of the bromodomain and extra terminal domain (BET) family, directly binds to the PD-L1 promoter and is significantly connected with PD-L1 expression [29]. The BET inhibitor JQ1 can inhibit PD-L1 mRNA and protein expression in lymphomas and leukemia mouse models [26, 30]. Mechanistically, JQ1 can reduce the binding of BRD4 to the PD-L1 promoter. In contrast, IFN-γ can enhance BRD4 binding to the PD-L1 promoter in ovarian cancer (OC) cells [31]. Similarly, BRD4 and interferon regulatory factor 1 (IRF1) can modulate the PD-L1 transcription triggered by interferon [30, 31].

## STATs

IFN-γ-induced PD-L1 expression promotes cancer immune escape, and this has been found in multiple tumor types. IFN-γ binds to the type II interferon receptor and activates janus kinase (JAK)-STAT signaling, further activating interferon response factors [32, 33]. For example, in CHL and primary mediastinal large B-cell lymphoma (PMBCL), gp24.1 amplification increases PD-L1 expression and its induction by the JAK2-STAT1-IRF1 signaling pathway [33]. Moreover, in melanoma, epigallocatechin gallate decreases PD-L1 expression by restraining STAT1 gene expression and phosphorylation followed by downregulation of IRF1 expression [34]. The JAK inhibitor JAKi was used in HNSCC cells to block STAT1 phosphorylation, thereby inhibiting the increase in PD-L1 mRNA levels induced by combined 5-FU and IFN-γ treatment [35]. The expression of inflammatory macrophages can be further increased by IFN-γ through elevating PD-L1 on these cells in a STAT1-dependent manner [36]. The STAT protein family member STAT3 binds directly to the PD-L1 promoter to activate transcription. Chimeric nucleophosmin (NPM)/anaplastic lymphoma kinase (ALK) is significantly associated with malignant cell transformation and NPM/ALK-carrying T cell lymphoma (ALK + TCL) cells abundantly express PD-L1, which is regulated by STAT3, and knocking down STAT3 can inhibit this effect. [37]. Under hypoxia, p-STAT3 interacts with PD-L1 to promote its nuclear translocation and enhances gasdermin C (GSDMC) transcription, thereby inducing pyroptosis in breast cancer (BC) cells [38]. In Epstein-Barr virus (EBV)-positive nasopharyngeal carcinoma cell lines, PD-L1 expression is greater, and latent membrane protein 1 (LMP1) activates PD-L1 by increasing STAT3 phosphorylation [39]. Conversely, water extract from sporoderm-broken spores of G. lucidum (BSGWE) can reduce PD-L1 expression in osteosarcoma by blocking STAT3 phosphorylation [40]. By downregulating CD40 and STAT3 expression, miR-502-5p can downregulate PD-L1 expression in gastric cancer (GC) cells [41].

## NF-κB

NF-κB is a homologous or heterologous dimer formed by members of the Rel family, and it plays a key role in cell growth, apoptosis, and immune responses through transcriptional regulation. LMP1 may induce PD-L1 expression in natural killer/T-cell lymphoma cells through regulation of the MAPK/NF-KB signaling pathway and is associated with poor prognosis [42]. IFN-γ stimulates NF-κB in melanoma cells to induce the expression of PD-L1, and inhibiting NF-κB expression lowers IFN-γ-induced PD-L1 but not constitutive PD-L1 expression [43]. In prostate cancer, RelB, a major member of the NF-κB family, upregulates PD-L1 mainly by binding to the NF-κB element located in the promoter of PD-L1 [44]. Furthermore, knockdown of histone deacetylase 5 (HDAC5) results in notable activation of NF-κB signaling, thus significantly increasing PD-L1 expression in pancreatic cancer (PC) [45].

## CDK5

CDK5 is a cyclin-dependent kinase that is widely expressed in many malignancies, allowing cancer cells to escape immune detection. The CDK5 inhibitor roscovitine prevents IFN-induced PD-L1 expression in medulloblastoma. Inhibition of CDK5 causes the PD-L1 transcriptional repressors IRF2 and IRF2BP2 to compete with IRF1 for binding to regions in the promoter of PD-L1 [46].

## HIF-1α

HIF-1α is a master regulator of the response to hypoxia via its activation of the transcription of many genes. Under hypoxic conditions, there was a positive correlation between the HIF-1α and PD-L1 expression levels together with the inactivation of T cells. In addition to proliferation and inhibition of apoptosis, HIF-1α has an immunosuppressive function [47, 48]. HIF-1α and PD-L1 expression levels were positively correlated with T cell inactivation under hypoxic circumstances, and inhibiting PD-L1 expression improved bone marrow-derived suppressor cells (MDSCs)-mediated T cell activation [49]. The combination of the HIF-1α inhibitor PX-478 and an anti-PD-L1 antibody increased dendritic cell (DC) and CD8 + T cell activation and dramatically suppressed tumor growth in a glioma mouse model. Mechanistically, HIF-1α activates PD-L1 transcription by binding to the hypoxia response element HRE-4 in the PD-L1 proximal promoter [50, 51]. In both acute myeloid leukemia (AML) mouse model and AML cell lines including MOLM-13 and THP1, PD-L1 boosts glycolytic metabolism, which enhances cell proliferation and tumor formation via the Akt/mTOR/HIF-1α signaling pathway [52].

## NRF2

NRF2 is a crucial transcription factor for antioxidant and detoxification enzymes that protect cells from damage caused by oxidative stress [53]. This protein decreases the expression of miR-1, leading to the elevation of PD-L1 in HCC patients treated with sorafenib [22]. The management of colon cancer has been transformed by the development of ICIs. Nonetheless, aberrant upregulation of PD-L1 promotes chemoresistance and results in poor prognosis, while NRF2 inhibition remarkably lowers PD-L1 mRNA expression in sensitive and resistant cells [54].

## ATF3

ATF3 is a common stress-inducible transcription factor that is instrumental in modulating oncogenesis, immunity, and metabolism. In melanoma and NSCLC cells, adenosine A1 receptor (ADORA1) inhibition promotes PD-L1 gene expression through ATF3 binding to the PD-L1 promoter directly. Similarly, Sophora alopecuroides Linn impedes PD-L1 expression by enhancing ADORA1 activation in NSCLC [55, 56].

In addition to the above-mentioned, directly regulating transcription factors, some genes such as phosphatase and tensin homolog (PTEN), mTOR, and p53, which are closely related to tumor development, can indirectly regulate PD-L1 transcription and thus promote tumor immune escape [52, 57, 58]. In conclusion, the above transcription factors regulate PD-L1 expression, influence tumor immune escape, and facilitate tumor progression and the development of drug resistance.

## Epigenetic regulation of PD-L1

DNA methylation, histone modification, non-coding RNA–mediated regulation, and N6-methyladenosine are the most common epigenetic regulation mechanisms. Epigenetic regulation is important for modulating several cellular functions, including PD-L1 expression (Fig 2).

## DNA methylation

DNA methylation occurs when a methyl group is added to the C5 position of a DNA cytosine loop to produce 5-methylcytosine (5mC), and it regulates gene expression by controlling chromatin structure and DNA stability and conformation [59]. Recent research has linked DNA methylation of the PD-L1 promoter to PD-L1 mRNA expression in various malignancies [60–63]. PD-L1 expression can be controlled by DNA methylation, and when its promoter is hypermethylated, it improves the overall survival of melanoma patients [62, 64]. Furthermore, the demethylating drug decitabine dose-dependently increases PD-L1 mRNA levels in leukemia cells [39]. Thus, inhibitors of epigenetic regulation may improve the effectiveness of anti-PD-1/PD-L1 antibodies.

## Histone modification

Recently, histone modification, particularly histone acetylation, has also been implicated in the transcriptional regulation of PD-L1. TET2, a ten-eleven translocation (TET) family member, recruits histone deacetylases (HDAC1/2) to deacetylate the histone modification H3K27ac on the PD-L1 promoter and consequently restraining PD-L1 transcription in BC [53]. HDAC3 overexpression suppressed PD-L1 and inhibited CD3^+^ T cell proliferation [65]. Suberoylanilide hydroxamic acid (SAHA), an HDAC inhibitor, reduced PD-L1 expression in lung cancer cells in a dose-dependent manner [66]. These investigations suggest combining HDAC inhibitors with PD-1/PD-L1 inhibitors may improve therapeutic efficacy.

Apart from histone acetylation, histone methylation in the PD-L1 promoter can influence PD-L1 transcription. The histone methyltransferase EZH2 represses PD-L1 transcription by histone H3 lysine 27 trimethylation (H3K27me3) of the PD-L1 promoter [67]. In contrast, the histone methyltransferase lysine methyltransferase 2 A (MLL1) promotes PD-L1 transcription through histone H3 lysine 4 trimethylation (H3K4me3) of the PD-L1 promoter [68].

## MicroRNAs

MicroRNAs (miRNAs) are a type of non-coding, single-stranded RNA molecules. Some miRNAs can bind to the 3’UTR of PD-L1 mRNA (shown in Table 1) e.g., miR-34 and miR-513, to degrade PD-L1 mRNA or inhibit PD-L1 translation [69–71]. In NSCLC, p53 increases miR-34 expression to reduce PD-L1 expression. MiR-34 can bind directly to the 3’UTR of PD-L1 mRNA to suppress PD-L1 production and antagonize T cell exhaustion [58]. Unlike miR-34, miR-197 indirectly represses PD-L1 expression via CDC28 protein kinase regulatory subunit 1B (CKS1B)/STAT3 in chemotherapy-resistant NSCLC [72].

**Table 1:** MicroRNAs were demonstrated to modulate PD- L1 in cancer cells in recent research

microRNA	cancer type	Efx	targets	mechanisms	Refs.
miR-138-5p	CC	↓	PD-L1		[161]
miR-148a-3p		↓	PD-L1		[162]
miR-15a		↓	HOXC4	MiR-15a inhibits PD-L1 expression via binding to homeobox C4 (HOXC4).	[163]
miR-20b-21, miR-130b		↑	PTEN	MiR-20b, -21, and − 130b promote PD-L1 expression via restraining PTEN.	[164]
miR-152	GC	↓	PD-L1		[165, 166]
miR-16-5p		↓	PD-L1		[167]
miR-200b		↓	PD-L1		[166]
miR-140	NSCLC	↓	PD-L1		[168]
miR-377-3p		↓	PD-L1		[169]
miR-34		↓	PD-L1		[58]
miR-3127-5p		↑	STAT3	MiR-3127-5p induces upregulation of PD-L1 expression by regulating the expression of p-STAT3.	[170]
miR-197		↓	CKS1B	MiR-197/CKS1B/STAT3 inhibits PD-L1 expression.	[171]
miR-155-5p	LUAD	↓	PD-L1		[172]
miR-320a	MM	↓	PD-L1		[173]
miR-let-7b	LC	↓	PD-L1		[174]
miR-4759	BC	↓	PD-L1		[175]
miR-27a-3p		↑	MAGI2	MiR-27 A-3p upregulates PD-L1 via the membrane-associated guanylate kinase inverted 2 (MAGI2) /PTEN/PI3K axis and together promotes immune escape from BC.	[176]
miR-92		↑	LATS2	MiR-92 binds to large tumor suppressor kinase 2 (LATS2) which is capable to interact with Yes1 associated transcriptional regulator (YAP1) to promote PD-L1 expression.	[177]
miR-424	OC	↓	PD-L1		[178]
miR-142-5p	PC	↓	PD-L1		[179]
miR-129-5P	DLBCL	↓	ARID3A	MiR-129-5p inhibits AT-rich interaction domain 3 A (ARID3A) and thereby downregulates PD-L1 expression.	[180]
miR-329-3p	HCC	↓	KDM1A	MiR-329-3p inhibits PD-L1 expression and enhances response to T cell-induced HCC cytotoxicity by targeting lysine-specific demethylase 1 A (KDM1A).	[181]
miR-155	Multiple cancers	↓	PD-L1		[182]
miR-200		↓	PD-L1		[71]
miR-let-7a/c/e		↓	PD-L1		[183]

The up and down arrows represent the up- and down-regulation of PD-L1, respectively.

*CC* colorectal cancer,* GC* gastric cancer,* NSCLC* non-small-cell lung cancer,* LUAD* lung adenocarcinoma,* MM* malignant mesothelioma,* LC* lung carcinogenesis,* BC* breast cancer,* OC* ovarian cancer,* PC* pancreatic cancer,* DLBCL* diffuse large B cell lymphoma,* HCC* hepatocellular carcinoma

**Table 2:** Regulators of PD-L1

Type	Regulators of PD-L1	Cancer type
Transcription factors	MYC	↑T-ALL[26], NSCLC[27]
	BRD4	↑lymphomas and leukemia[26, 30], OC[31]
	STAT3	↑T cell lymphoma[37], BC[38], osteosarcoma[40], GC[41]
	STAT1	↑CHL and PMBCL[33], melanoma[34], HNSCC[35]
	NF-κB	↑prostate cancer [44], PC [45]
	CKD5	↑medulloblastoma [46], melanoma[32]
	HIF-1α	↑multiple cancers[47, 48] [184]
	MLL1	↑PC[134]
	NRF2	↑HCC[22]
	ATF3	↑melanoma and NSCLC[55]
Epigenetic regulation of PD-L1	DNA methylation	↓melanoma[62, 64], leukemia [39]
	Histone modification	↓BC and B cell lymphomas[53, 185], lung cancer[66]
	MicroRNAs	shown in Table 1
	N6- methyladenosine	↑OSCC[90], ↑BC[91]
	LncRNA and circRNA	HOTTIP, SNHG12, EMX2OS- ↑OC[76, 77, 81] SNHG20-↑esophagus cancer[78] CASC11-↑HCC[80] SNHG14-↑DLBCL[79] NUTM2A-AS1-↑GC[82] Hoxa-AS2-↑nasopharyngeal carcinoma[83] IFITM4P-↑OSCC[84] LncMX1-215-↓HNSCC[85] Hsa_circ_0000190-↑NSCLC[186] HasCircRNA-002178-↑ LUAD[87] CDR1-AS-↑CC[88]
PD-L1 regulation at the protein level	Ubiquitination	↓ BC[97], NSCLC[98], CC[99], colon cancer[100], melanoma[101, 102, 106], lung cancer[101], esophageal squamous cell carcinoma[105]
	Glycosylation	↑AML[108], TNBC[109, 111], colon cancer[110], prostate cancer[111], glioma[112]
	Phosphorylation	↓ BC[114, 116], HCC[115]
	Acetylation	↓ multiple cancers[117]
	Palmitoylation	↑ BC and colon cancer[118, 119]
	Autophagy	↓ OC[122]

The up and down arrows represent the up- and down-regulation of PD-L1, respectively.

**Table 3:** Drugs associated with PD-L1 regulation

Drug	Target	Cancer type	Model type
Stri-201	STAT3-PD-L1	↓HNSCC[130]	mouse
Nexturastat Tubastatin A	HDAC6-STAT3-PD-L1	↓melanoma[131]	cell
Silvestrol	EIF4F-STAT1-PD-L1	↑melanoma[132]	mouse
Verteporfin	STAT1-IRF1-TRIM28	↓multiple cancers[133]	cell
Verticillin A	MLL1- H3K4me3	↓PC[134]	cell
Cisplatin	miR-145- c-MYC-PD-L1	↑ovarian carcinoma[135]	cell
PROTACs	PD-L1 ubiquitination and lysosomal degradation	↓multiple cancers[138, 139]	mouse
PD-LYSO	HIP1R-PD-L1	↓multiple cancers[140]	cell
Crcumin	CSN5-PD-L1	↓BC[97]	mouse
Decitabine	DNA hypomethylation	↑CC, leukemia, HNSCC, NSCLC [60, 142–144]	mouse
Temozolomide	STAT3-PD-L1	↑glioblastoma multiforme[145]	mouse
Regorafenib	RET-Src-JAK1/2-STAT1; RET-Src- MAPK signaling	↑melanoma[146]	mouse
Mifepristone	glucocorticoid receptor-PD-L1	↓PC[147]	mouse
Metformin	PD-L1 glycosylation	↓breast tumor, melanoma, and colon cancer[109, 148]	mouse
Capmatinib	MET-PD-L1	↓PC[149]	mouse
Albendazole	PD-L1 ubiquitination	↓melanoma[101]	mouse

The up and down arrows represent the up- and down-regulation of PD-L1, respectively.

**Table 4:** Clinical efficacy of FDA-approved PD-L1 inhibitors

PD-L1 inhibitors	Cancer type	Trial (Phase)	Patients(n)	ORR (%)	mPFS (months)	mOS (months)
Avelumab	Metastatic UC[151]	Ib	44	18.2	2.9	13.7
	RCC[152]	III	886	55.2	13.8	11.6
	MCC[153]	II	88	33.0	-	12.6
Atezolizumab	MUC[155]	II	310	26.0	2.1	11.4
	NSCLC[154]	III	1021	6.3	13.5	-
Durvalumab	NSCLC[156]	III	713	-	14.5	25.2
	TNBC with PD-L1(+)[157]	II	199	-	-	27.3
Cemiplimab	CSCC[158]	II	78	44	-	-
	NSCLC[159]	I	20	25.0	-	-

*ORR* objective response rate,* mPFS* median progression-free survival,* mOS* median overall survival

## LncRNA and circRNA

Long non-coding RNA (lncRNA) and circular RNA (circRNA) are crucial types of ncRNAs that are sensitive to the tumor immune response. LncRNAs are involved in a variety of cellular processes and molecular signaling cascades via their modulation of PD-L1 expression at the epigenetic, transcriptional, and post-transcriptional levels [73, 74]. Some circRNAs regulate the function of miRNAs as microRNA sponges and are essential to transcriptional regulation [75].

The lncRNA HOTTIP stimulates neutrophil IL-6 secretion to allow STAT3 phosphorylation, which increases PD-L1 expression in OC, limiting T cell activity and eventually accelerating tumor immune escape [76]. By interrupting miR-21/IL-6 crosstalk, the lncRNA small nucleolar RNA host gene 12 (SNHG12) promotes the PD-L1 expression regulated by IL-6R [77]. Compared with SNHG12, inhibition of the lncRNA SNHG20 downregulates PD-L1 expression through the ATM/JAK/PD-L1 pathway, affecting the epithelial-mesenchymal transition (EMT) and metastasis in esophageal cancer [78]. In DLBCL, the lncRNA SNHG14 increases the expression of zinc finger E-box binding homeobox 1 (ZEB1) by sponging miR-5590-3p. Subsequently, SNHG14 and PD-L1 are transcriptionally activated by ZEB1, thereby promoting tumor immune evasion [79]. Through EIF4A3-mediated E2F transcription factor 1 (E2F1) upregulation, the lncRNA cancer susceptibility candidate 11 (CASC11) inhibited NF-κB and PI3K/AKT/mTOR pathway activation to mediate PD-L1 expression and encourage tumor progression in a mouse model of HCC with lung metastasis [80]. In OC, the EMX2OS/miR-654/AKT3/PD-L1 axis is associated with aggressiveness, and PD-L1 upregulation reverses the anti-cancer functions of miR-654. miR-654 is sponged and downregulated by lncRNA EMX2OS, while, in contrast, AKT3 and PD-L1 are upregulated. [81]. The lncRNA NUTM2A-AS1 directly targets miR-376a, which increases TET1 and HIF-1α expression followed by an increase in PD-L1 expression, thereby promoting gastric carcinogenesis and drug resistance [82]. Similarly, Hoxa-AS2 binds to miR-519 and results in significant upregulation of HIF-1α and PD-L1, which prominently promotes the progression of nasopharyngeal carcinoma including its proliferation, migration, and invasive ability [83]. In oral squamous carcinoma (OSCC), the lncRNA IFITM4P enhances the binding of histone 3 lysine 4 demethylase KDM5A to the PTEN promoter to diminish PTEN transcription, boosting PD-L1 expression. It also recruits SAM and SH3 domain containing protein 1 (SASH1) in the cytoplasm to bind and phosphorylate TAK1 (Thr187), increasing NF-κB phosphorylation to stimulate PD-L1 transcription [84]. Conversely, the expression of PD-L1 is extremely hindered by LncMX1-215 through the blocking of GCN5-mediated acetylation of H3K27 [85].

Hsa_circ_0000190 enhances PD-L1 mRNA-mediated expression of soluble PD-L1 and promotes non-small cell lung carcinogenesis and immune evasion [86]. In addition, HasCircRNA-002178 is capable of enhancing PD-L1 expression by inducing T cell exhaustion via sponging miR-34 in LUAD cells [87]. CDR1-AS remarkably increases the expression of CMTM4 and CMTM6, the pivotal regulators of PD-L1 protein, and concurrently upregulates PD-L1 expression [88] (Table 2).

## N6-methyladenosine

N6-methyladenosine (m6A) is a common mRNA modification that regulates mRNA stability, localization, transport, shearing, and translation. METTL3 mediates the RNA methylation modification process and functionally catalyzes m6A mRNA methylation [89]. By increasing the m6A content in protein arginine methyltransferase 5 (PRMT5) and PD-L1, METTL3 promotes the metastasis and proliferation of OSCC [90]. Through a m6A-dependent mechanism, METTL3 positively regulates IGF2BP3 to increase the stability and expression of PD-L1 mRNA in BC [91]. YTH domain family proteins (YTHDF) recognize and bind m6A in mRNA, YTHDF1 mediates translation to promote translation efficiency, and YTHDF2 mediates degradation to control the half-life of target transcripts, ensuring efficient protein production from m6A-tagged dynamic transcripts [92]. Recent evidence suggests that m6A demethylases fat mass and obesity-associated protein (FTO) and ALKBH5 induce m6A mRNA demethylation [93]. ALKBH5 deficiency enhances m6A modification in the PD-L1 3’UTR region, thereby promoting PD-L1 mRNA degradation in a YTHDF2-dependent manner [94]. Conversely, FTO promotes PD-L1 expression by inducing m6A demethylation of mRNA in colon cancer cells; however, whether m6A demethylase acts directly on PD-L1 mRNA remains uncertain [95] (Table 3).

## PD-L1 regulation at the protein level

The ultimate mechanism regulating PD-L1 expression is post-translational regulation, which is influenced by ubiquitination, phosphorylation, glycosylation, acetylation, palmitoylation, autophagy, and other factors (Fig 2).

## Ubiquitination

Ubiquitination is a common post-translational modification that controls the stability of proteins [96]. TNFα secreted by macrophages can positively regulate PD-L1 protein at the post-translational level without affecting its transcription. TNFα activates NF-κB through the nuclear translocation and downstream transactivation of p65. Subsequently, p65 activates COP9 signalosome 5 (CSN5) transcription and promotes CSN5 expression. CSN5 binds PD-L1 and deubiquitinates PD-L1, enhancing PD-L1 stability, and evading T cell immune surveillance [97]. Additionally, berberine binds to CSN5 and diminishes its deubiquitination activity, leading to PD-L1 ubiquitination and degradation and promoting anti-tumor immunity [98]. LPS or high-cholesterol diet (HCD)-induced macrophage infiltration significantly activates the C-C motif chemokine ligand 5 (CCL5)-P65/STAT3-CSN5-PD-L1 signaling pathway in azoxymethane-induced CC mouse models and is correlated with poor prognosis [99]. In contrast to CSN5, the deubiquitinase USP8 removes TNF receptor associated factor 6 (TRAF6)-mediated K63-linked ubiquitination, thereby promoting PD-L1 degradation in mouse models of lung and colon cancer [100]. Ubiquilin 4 (UBQLN4) suppresses PD-L1 ubiquitination and promotes protein stability in melanoma. Albendazole stimulates tumor immune function by reducing UBQLN4 expression and, as a result, facilitating PD-L1 protein degradation [101]. Overexpression of the E3 ligase ITCH in melanoma cells ubiquitinates and suppresses MAPK-induced PD-L1 expression, boosts CD8 + cell production, and promotes antitumor effects [102]. The CMTM family has a significant impact on the immune system and is involved in the occurrence and development of tumors. A quintessential example is that EMT transcription factor SNAI1 promotes PD-L1 expression in BC by positively regulating CMTM6 [103]. Two groups used whole-genome CRISPR–Cas9 screening and haploid gene screening based on the fluorescence-activated cell sorting technology to jointly discover that CMTM6 is a key protein that modulates PD-L1 stability. CMTM6 interacts with PD-L1 and co-localizes at endosomes and the plasma membrane. CMTM6 protects PD-L1 from lysosomal-mediated degradation, increases its stability, and enhances the ability of tumor cells to suppress immune responses. Inhibiting CMTM6 expression can diminish PD-L1 expression and greatly limit the tumor cell’s ability to block T cell activity, but it has little effect on the MHC class I molecules [104]. Mechanistically, CMTM6 inhibits the ubiquitination of PD-L1 and prolongs its half-life. In tumor cells, CMTM6 is involved in BCLAF1-dependent PD-L1 upregulation through inhibition of ionizing radiation-induced PD-L1 ubiquitination [105]. Similar to CMTM6, CMTM4 also functions to regulate PD-L1 and is an alternative regulator of this protein [106] (Table 4).

## Glycosylation

Glycosylation of membrane receptor proteins influences not only the interaction of ligands and receptors but also protein activity [107]. In human tumor tissues and cancer cell lines, PD-L1 is glycosylated. Glycogen synthase kinase 3β (GSK3β) is associated with non-glycosylated PD-L1 and triggers PD-L1 degradation by β-TrCP. PD-L1 N192, N200, and N219 glycosylation can antagonize GSK3β binding, thereby increasing the stability of PD-L1 [108]. Similarly, the N-glycosyltransferase subunit STT3 can increase the glycosylation and stability of PD-L1, resulting in cancer cells evading the immune system [109, 110]. Moreover, a spliced isoform, FKBP51s, and sigma-1 receptor, SIGMAR1, increased PD-L1 expression by promoting PD-L1 glycosylation and stability [111, 112].

## Phosphorylation

Non-glycosylated PD-L1 is an unstable protein whose T180 and S184 residues are easily phosphorylated by GSK3β and then bound by the E3 ubiquitin ligase β-TrCP, resulting in PD-L1 degradation in the cytoplasm [113]. For instance, in basal-like breast cancer, suppression of GSK3β activity by epidermal growth factor stabilizes PD-L1 [114]. IL-6-activated JAK1 phosphorylates PD-L1 Tyr112 and recruits the N-glycosyltransferase STT3A, which catalyzes PD-L1 glycosylation and stabilizes PD-L1 [115]. Another important player regulating PD-L1 phosphorylation is AMP-activated protein kinase (AMPK). By directly phosphorylating S195 of PD-L1 in response to metformin activation of AMPK, aberrant PD-L1 glycosylation is induced, which causes endoplasmic reticulum accumulation and ER-related destruction [116].

## Acetylation and palmitoylation

Acetylation is a common post-translational modification of proteins. PD-L1 is acetylated by p300 enzyme at Lys263 affecting nuclear translocation. HDAC2 enzyme catalyzes PD-L1 deacetylation and binds to huntingtin interacting protein 1 related (HIP1R), thereby translocating to the nucleus. Accumulation of nuclear PD-L1 may promote tumor cells to evade immune surveillance during metastasis [117]. Palmitoyltransferase ZDHHC3 (DHHC3) catalyzes PD-L1 palmitoylation in colon and breast malignancies and stabilizes PD-L1 by suppressing ubiquitination, which prevents lysosomal degradation of PD-L1. Using 2-bromopalmitate, the suppression of PD-L1 palmitoylation or the silencing of DHHC3 triggers anticancer immunity [118, 119].

## Autophagy

Autophagy is a membrane transport process involved in the metabolism of intracellular component degradation that is activated under nutrient or energy deprivation conditions. Under adverse conditions, such as hypoxia, a shortage of growth hormones, or reactive oxygen species (ROS), autophagy also allows cells to survive [120, 121]. With an in-depth study of autophagy in the tumor immune response, the relationship between PD-L1 and autophagy in tumors has been explored. PD-L1 upregulates beclin 1 (BECN1), a crucial molecule in autophagy regulation, and enhances the autophagy of OC cells [122]. Interestingly, IFN-γ promotes PD-L1 expression by inhibiting autophagy via p62/ sequestosome 1 (SQSTM1) accumulation and NF-κB activation [123] (Table 2).

## Targeting PD-L1 and the PD-L1 regulatory pathway for cancer immunotherapy

Cancer immunotherapy involving PD-1/PD-L1 blocking antibodies has brought considerable therapeutic advantages to patients with advanced-stage cancer [124]. Research on anti-PD-L1 antibodies has been conducted in a variety of tumors, including NSCLC, SCLC, melanoma, HCC, BC, head and neck squamous cell carcinoma, gastric and gastroesophageal junction cancer, OSCC, urothelial carcinoma (UC), renal cell carcinoma (RCC) [125]. FDA-approved anti-PD-1/PD-L1 applications are quickly expanding across various tumor types. The PD-L1 level in tumors is an essential factor influencing the therapeutic efficacy of anti-PD-1/PD-L1 therapy [126, 127]. The combination of PD-L1 and PD-1 results in the loss of the killing ability of tumor-infiltrating lymphocytes, which leads to uninhibited tumor growth. Therefore, the destruction of the interaction between PD-L1 and PD-1 manifests tremendous potential in releasing the lethality of the immune system to cancer cells [128, 129]. Here, we summarize the research progress of small-molecule agents that target the PD-L1/PD-1 axis and PD-L1 inhibitors (Table 3).

Numerous small molecule agents targeting epigenetic regulation directly or indirectly downregulate PD-L1 and increase immunotherapy effects. Notably, The STAT family is critical in the control of PD-L1. For instance, Stri-201, a small molecule inhibitor targeting STAT3, can effectively inhibit the expression of PD-L1 in the human tongue squamous cell carcinoma cell line CAL27 cells [130]. Nexturastat and tubastatin A inhibited HDAC6-mediated STAT3 activity in melanoma, leading to PD-L1 expression reduction [131]. The EIF4F inhibitor silvestrol enhances IFN-γ-induced PD-L1 transcription and elevates anti-tumor immunomodulatory effects in melanoma [132]. Contrary to silvestrol, verteporfin blocks the STAT1-IRF1- tripartite motif containing 28 (TRIM28) signaling cascade and induces autophagy-mediated degradation of the Golgi apparatus, thereby efficiently downregulating PD-L1 expression [133]. By impairing MLL1, the epipolythiodioxopiperazine metabolite verticillin A lowers the amount of H3K4me3 in the PD-L1 promoter, transcriptionally hindering PD-L1 expression and enhancing the effectiveness of PD-L1/PD-1 immunotherapy in PC patients [134]. By targeting c-MYC, cisplatin inhibits miR-145 expression, which results in upregulation of PD-L1 in OC [135]. Transforming growth factor-beta (TGF-β) affects PD-L1 inhibitors to some extent and induces drug resistance. YM101 can suppress TGF-β and PD-L1, which dramatically enhances the anti-tumor effect [136]. Furthermore, Mn^2+^ acts synergistically with YM101 to facilitate the conversion of non-inflammatory to immune inflammatory tumors and greatly antagonizes PD-1/PD-L1 drug resistance [137].

Post-translational modifications are critical to PD-L1 stability; therefore, targeting this process may lead to irreversible PD-L1 degradation. Proteolysis targeting chimeras (PROTACs) induce PD-L1 ubiquitination and subsequent lysosomal degradation by recruiting E3 ligases. PROTACs can induce PD-L1 protein degradation in various malignant cells in vivo in a proteasome-dependent manner [138, 139]. Similarly, the peptide PD-LYSO, which contains the lysosome-sorting signal as well as the PD-L1-binding sequence of HIP1R, causes PD-L1 expression to be reduced in tumor cells [140]. CSN5 is required for PD-L1 stabilization owing to its inhibition of the ubiquitination of PD-L1. Thus, the CSN5 inhibitor curcumin increases tumor cell susceptibility to CTLA4 therapy by lowering PD-L1 expression [97]. Protein kinase AMPK agonists or ketogenic diets promote PD-L1 phosphorylation and disrupt its interaction with CMTM4, which contributed to inducing PD-L1 degradation and enhancing anti-CTLA4 immunotherapy in mouse tumor models [141].

Some chemotherapy drugs are closely correlated with PD-L1 regulation. For instance, the DNA demethylation drug decitabine induced DNA hypomethylation in CC, directly upregulated PD-L1 expression, stimulated more antitumor effects, and significantly enhanced the immunotherapeutic effects of PD-L1 [142]. This phenomenon was similarly observed in mouse models of leukemia, HNSCC, and NSCLC [60, 143, 144]. Temozolomide can trigger STAT3 activation, subsequently increasing PD-L1 expression [145]. In melanoma, regorafenib inhibits JAK1/2-STAT1 and MAPK signaling by targeting the RET-Src axis, consequently attenuating IFN-γ-induced PD-L1 expression [146]. A previous study has reported that the glucocorticoid receptor inhibitor mifepristone and dual ICB act synergistically in PC to inhibit PD-L1 expression while promoting cytotoxic T cell infiltration and activity, enhancing antitumor immunity [147]. Metformin activates AMPK, which phosphorylates S195 of PD-L1, resulting in abnormal PD-L1 glycosylation [109]. In contrast, metformin reduces the abundance of PD-L1 by disrupting electrostatic interactions, thereby promoting the dissociation of the cytoplasmic structural domain membrane of PD-L1 [148]. Aberrant activation and expression of receptor tyrosine kinases (RTKs) are relevant to numerous human cancers. MET was confirmed as a specific RTK in PC, and it is abundant in PC tissues and positively correlates with PD-L1 levels. Notably, the MET inhibitor capmatinib has a pivotal function in restraining PD-L1 expression and stopping tumor progression [149]. By inhibiting UBQLN4 and promoting ubiquitination in melanoma cells, Albendazole has an anti-tumor immunological impact, culminating in PD-L1 protein degradation [101].

The majority of the small molecule drugs mentioned above target not just PD-L1, but also additional proteins that influence tumor survival, proliferation, and metabolism. This gives them advantages over PD-L1 antibodies. Small molecule medications, however, may also activate alternate pathways or feedback mechanisms that control PD-L1 expression, producing ineffective anti-tumor effects. Consequently, concurrently in-depth comprehension of PD-L1 antibodies is still required. There are currently four types of PD-L1 inhibitors approved by the FDA, including Avelumab, Atezolizumab, Durvalumab, and Cemiplimab (Table 4). Avelumab (MSB0010718C) is a fully human IgG1 monoclonal antibody that can mediate ADCC by targeting PD-L1 [18]. It is effective in clinical trials for treating metastatic merkel cell carcinoma (MCC), metastatic UC, RCC and is well tolerated by patients [150–153]. Remarkably, Avelumab was the first drug licensed by the FDA for the treatment of MCC. Currently, atezolizumab (MPDL3208A), a humanized human and murine cross-reactive therapeutic PD-L1 antibody, is in clinical trials for patients with NSCLC and locally advanced and metastatic urothelial carcinoma [154, 155]. Durvalumab is a PD-L1-targeting immunosuppressant that the European Medicines Agency approved for the consolidation of locally advanced PD-L1-positive NSCLC following chemoradiotherapy [156]. While maintenance chemotherapy is more successful than duvacizumab in patients with hormone receptor-positive and HER2-negative breast cancer, duvacizumab increases overall survival (OS) in PD-L1-positive TNBC[157]. Cemiplimab is presently approved by the FDA for the treatment of patients with metastatic cutaneous squamous cell carcinoma (CSCC) or locally advanced unresectable CSCC and NSCLC [158, 159].

## Conclusions

Cancer immunotherapy with PD-1/PD-L1 blocking antibodies has ushered in a new era of cancer treatment, but only a fraction of patients have shown objective clinical responses. Currently, FDA-approved PD-L1 inhibitors are indicated for locally advanced or metastatic UC, NSCLC, MCC, and CSSS. PD-L1 expression in tumors, microsatellite instability, tumor mutational burden, and tumor-infiltrating lymphocytes are biomarkers that indicate how well PD-1/PD-L1 inhibitors work [160]. Treatment with small-molecule drugs that modulate PD-L1 expression is a favorable strategy for ​​cancer therapy; nevertheless, it can be a double-edged sword. On the one hand, induction of PD-L1 expression in tumor cells may increase the sensitivity of cancer cells to PD-1/PD-L1 immune checkpoint blockade, such as Poly-(ADP-ribose) polymerase (PARP) inhibitors in breast cancer and MET proto-oncogene, receptor tyrosine kinase (MET) inhibitors in HCC. Nevertheless, inducing PD-L1 expression artificially may also promote immunosuppression. On the other hand, the combination of PD-L1-targeting small-molecule drugs with anti-PD-1/PD-L1 antibodies may produce synergistic anticancer activity, but it may also render the antibodies ineffective due to loss of immune checkpoint expression. As noted in this review, the expression of PD-L1 is governed by a number of regulatory mechanisms. Transcription factors, oncogenes, tumor suppressor genes, and microRNAs all regulate PD-L1, affecting anti-tumor immune responses. Selecting one or the other to alter immune checkpoint expression may be inefficient, as other regulatory elements may compensate and become overactivated. Therefore, further preclinical and clinical studies will be needed to advance the understanding of tumor immune evasion mechanisms and the search for optimal combination therapies.

In conclusion, understanding the underlying regulatory pathways will improve current immunotherapies by manipulating PD-L1 expression.

## Data Availability

Not applicable.

## Abbreviations

PD-1: Programmed cell-death protein 1
PD-L1, CD274: Programmed cell death 1 ligand 1
ICs: Immune-checkpoint
CTLA4: Cytotoxic T-lymphocyte associated protein 4
LAG3: Lymphocyte activating 3
HAVCR2, TIM3: Hepatitis A virus cellular receptor 2
TIGIT: T cell immunoreceptor with Ig and ITIM domains
TILs: Tumor-infiltrating lymphocytes
ADCC: Antibody-dependent cell-mediated cytotoxicity
HNSCC: Head and neck squamous cell carcinoma
NSCLC: Non-small-cell lung cancer
PLAC8: Placenta-specific protein 8
NRF2: Nuclear factor E2-related factor 2
HCC: Hepatocellular carcinoma
BRD4: Bromodomain containing 4
NF-κB: Nuclear factor kappa-B
CDK5: Cyclin-dependent kinase 5
HIF-1α: Hypoxia inducible factor 1 subunit alpha
AFT3: Cyclic AMP-dependent transcription factor 3
T-ALL: T cell acute lymphoblastic leukemia
BIN1: Bridging integrator-1
CDK7: Cell cycle protein-dependent kinase 7
BET: Bromodomain and extra terminal domain
OC: Ovarian cancer
IRF1: Interferon regulatory factor 1
CHL: Classical Hodgkin lymphoma
PMBCL: Primary mediastinal large B-cell lymphoma
JAK: Janus kinase
EGCG: Epigallocatechin gallate
NPM: Nucleophosmin
ALK: Anaplastic lymphoma kinase
ALK + TCL: NPM/ALK-carrying T cell lymphoma
GSDMC: Gasdermin C
BC: Breast cancer
EBV: Epstein-Barr virus
LMP1: Latent membrane protein 1
BSGWE: Water extract of sporoderm-broken spores of G. lucidum
GC: Gastric cancer
MAPK: Mitogen activated kinase-like protein
PI3K: Phosphatidylinositol 3-kinase
HDAC5: Histone deacetylase 5
PC: Pancreatic cancer
MDSCs: Myeloid-derived suppressor cells
AML: Acute myeloid leukemia
mTOR: Mechanistic target of rapamycin kinase
ADORA1: Adenosine A1 receptor
PTEN: Phosphatase and tensin homolog
5mC: 5-methylcytosine
HDAC: Histone deacetylase
TET: Ten-eleven translocation
SAHA: Suberoylanilide hydroxamic acid
H3K27me3: Histone H3 lysine 27 trimethylation
MLL1: Lysine methyltransferase 2 A
H3K4me3: Histone H3 lysine 4 trimethylation
miRNAs: microRNAs
CKS1B: CDC28 protein kinase regulatory subunit 1B
HOXC4: Homeobox C4
MAGI2: Membrane-associated guanylate kinase inverted 2
LATS2: Large tumor suppressor kinase 2
YAP1: Yes1 associated transcriptional regulator
ARID3A: AT-rich interaction domain 3 A
KDM1A: Lysine-specific demethylase 1 A
CC: Colorectal cancer
LUAD: Lung adenocarcinoma
MM: Malignant mesothelioma
LC: Lung carcinogenesis
DLBCL: Diffuse large B cell lymphoma
lncRNA: Long non-coding RNA
circRNA: Circular RNA
SNHG12: Small nucleolar RNA host gene 12
EMT: Epithelial-Mesenchymal Transition
ZEB1: Zinc finger E-box binding homeobox 1
E2F1: E2F transcription factor 1
CASC11: Cancer susceptibility candidate 11
OSCC: Oral squamous carcinoma
m6A: N6-methyladenosine
PRMT5: Protein arginine methyltransferase 5
YTHDF: YTH domain family proteins
FTO: Fat mass and obesity-associated protein
CSN5: COP9 signalosome 5
HCD: High-cholesterol diet
CCL5: C-C motif chemokine ligand 5
TRAF6: TNF receptor associated factor 6
UBQLN4: Ubiquilin 4
GSK3β: Glycogen synthase kinase 3β
AMPK: AMP-activated catalytic subunit alpha 1
HIP1R: Huntingtin interacting protein 1 related
ROS: Reactive oxygen species
BECN1: Beclin 1
SQSTM1: Sequestosome 1
UC: Urothelial carcinoma
TNBC: Triple-negative breast cancer
RCC: Renal cell carcinoma
TRIM28: Tripartite motif containing 28
TGF-β: Transforming growth factor-beta
PROTACs: Proteolysis targeting chimeras
RTKs: Receptor tyrosine kinases
MCC: Merkel cell carcinoma
OS: Overall survival
CSCC: Cutaneous squamous cell carcinoma
PARP: Poly-(ADP-ribose) polymerase
MET: MET proto-oncogene, receptor tyrosine kinase
ORR: Objective response rate
mPFS: Median progression-free survival
mOS: Median overall survival
